# Bioinformatics analysis to screen the key prognostic genes in ovarian cancer

**DOI:** 10.1186/s13048-017-0323-6

**Published:** 2017-04-13

**Authors:** Li Li, Shengyun Cai, Shengnan Liu, Hao Feng, Junjie Zhang

**Affiliations:** The Department of Obstetrics and Gynecology, First Affiliated Hospital, Second Military Medical University, Changhai Road No 168, Shanghai, 200433 People’s Republic of China

**Keywords:** Ovarian cancer, Cluster analysis, Key prognostic genes, Functional enrichment analysis, Multivariate survival analysis

## Abstract

**Background:**

Ovarian cancer (OC) is a gynecological oncology that has a poor prognosis and high mortality. This study is conducted to identify the key genes implicated in the prognosis of OC by bioinformatic analysis.

**Methods:**

Gene expression data (including 568 primary OC tissues, 17 recurrent OC tissues, and 8 adjacent normal tissues) and the relevant clinical information of OC patients were downloaded from The Cancer Genome Atlas database. After data preprocessing, cluster analysis was conducted using the ConsensusClusterPlus package in R. Using the limma package in R, differential analysis was performed to identify feature genes. Based on Kaplan-Meier (KM) survival analysis, prognostic seed genes were selected from the feature genes. After key prognostic genes were further screened by cluster analysis and KM survival analysis, they were performed functional enrichment analysis and multivariate survival analysis. Using the survival package in R, cox regression analysis was conducted for the microarray data of GSE17260 to validate the key prognostic genes.

**Results:**

A total of 3668 feature genes were obtained, among which 75 genes were identified as prognostic seed genes. Then, 25 key prognostic genes were screened, including *AXL*, *FOS*, *KLF6*, *WDR77*, *DUSP1*, *GADD45B*, and *SLIT3*. Especially, *AXL* and *SLIT3* were enriched in ovulation cycle. Multivariate survival analysis showed that the key prognostic genes could effectively differentiate the samples and were significantly associated with prognosis. Additionally, GSE17260 confirmed that the key prognostic genes were associated with the prognosis of OC.

**Conclusion:**

*AXL*, *FOS*, *KLF6*, *WDR77*, *DUSP1*, *GADD45B*, and *SLIT3* might affect the prognosis of OC.

## Background

Ovarian cancer (OC), which ranks seventh in incidence and eighth in mortality among tumors in women, is characterized by pelvic pain, bloating, loss of appetite, and abdominal swelling [1]. OC can mainly spread into the lining of bowel and abdomen, and lymph nodes, bladder, liver, and lungs [2]. Usually, the women with more ovulation have higher risk of OC, especially those who have not given birth, have earlier menstruation or later menopause [3]. OC usually has a poor prognosis and high mortality, and most cases are diagnosed at advanced stages as there lacks effective detection means [4]. In 2012, globally 239,000 women were diagnosed with OC and nearly around 152,000 women died of the disease. [3]. Thus, exploring the pathogenesis of OC and developing novel therapies are urgent.

In recent years, several studies have reported the molecular mechanisms of OC. For instance, Li et al. declared that chemokine receptor 4 (*CXCR4*) plays a critical role in cisplatin-based chemotherapy for patients with epithelial ovarian cancer (EOC) and can be seen as a prognostic factor [5]. Yes-associated protein 1 (*YAP*) contributes to cell growth and formation of OC both in vivo and in vitro, additionally, *YAP* and TEA domain family member 4 (*TEAD4*) may serve as prognostic markers and therapeutic targets for OC [6, 7]. Previous studies demonstrated that high Beclin 1 expression in protein level can be a prognostic factor of OC [8, 9]. Califano et al. deemed that obesity evaluated by Body Mass Index combined with high mobility group A2 (*HMGA2*) expression can be used for predicting poor prognosis in patients suffered from OC [10]. Forkhead box M1 (*FOXM1*) expression is reported to be participated in the development and progression of EOC, and *FOXM1* is a promising prognostic factor for overall survival and progression-free survival [11, 12]. However, there lacks a overall reveal of the key genes implicated in OC.

To identify the key genes associated with prognosis of OC, microarray data of primary OC tissues, recurrent OC tissues and adjacent normal tissues were obtained. Then, the samples were pre-classified into two groups, and key prognostic genes were screened. Followed by functional enrichment analysis, multivariate survival analysis was carried out to examine the overall influence of these genes on prognosis. Finally, the key prognostic genes were validated by an independent microarray data.

## Methods

### Data source and data preprocessing

Gene expression data of OC patients (dataset ID: TCGA_OV_exp_u133a) were downloaded from TCGA (The Cancer Genome Atlas, http://cancergenome.nih.gov/) database [13], meanwhile, the relevant clinical information were also obtained. The gene expression data, which were sequenced on the platform of Affymetrix HT Human Genome U133a microarray, included 568 primary OC tissues, 17 recurrent OC tissues, and 8 adjacent normal tissues. The data is level 3 data downloaded from TCGA, in which the expression level of all probes has been normalized. Based on the annotation platform, probes were then mapped into gene symbols. For multiple probes corresponded to a common gene symbol, their values were averaged and defined as the gene expression value.

### Cluster analysis and differential analysis

The variance of gene expression levels for each gene in the samples was calculated, and the gene with variance less than 20% of the total variance of all genes was removed. Meanwhile, the median of gene expression level for each gene in each sample was used as the statistical indicator, and then the gene with median less than 20% of the total median of all genes was eliminated. The expression levels of the genes with potential expression changes in each sample were performed centralization. To pre-classify the samples into two groups, cluster analysis was conducted using the ConsensusClusterPlus package [14] in R. Subsequently, the limma package (Linear Models for Microarray Analysis, http://www.bioconductor.org/packages/release/bioc/html/limma.html) [15] in R was utilized to perform differential analysis for each gene in the pre-classified samples, and the genes with *p-*value < 0.001 were identified as feature genes.

### Screening of stable feature genes and key prognostic genes

To obtain novel sample classification and feature genes, the expression levels of feature genes were iteratively used for the above cluster analysis and differential analysis. Then, the novel sample classification and feature genes were separately compared with the previous sample classification and feature genes. If both of them were inconsistent, the expression matrix of the novel feature genes would be applied for the next iteration. The stable feature genes were obtained until at least one of the sample classification and the feature genes was consistent. To further identify the stable feature genes associated with prognosis, the expression level of each gene in each sample was used to classify the samples according to the average expression level of the gene. Then, Kaplan-Meier (KM) survival analysis [16] was carried out, and the feature genes with *p-*value < 0.01 were taken as prognostic seed genes.

The prognostic seed genes were performed Euclidean distance cluster to reclassify the samples. Then, the reclassified samples were conducted KM survival analysis [16]. Based on this sample classification, the differential expression level of each prognostic seed gene was calculated using the limma package [15] in R, with *p-*value < 0.001 as the threshold. Log_2_ fold change (FC) value (with fixed interval) was used as the cut-off criterion for screening gene set. Then, cluster analysis and KM survival analysis [16] successively were performed, and the most significant gen were considered as the ultimately key prognostic genes.

### Functional enrichment analysis

Gene Ontology (GO, http://www.geneontology.org/) aims to provide functions of genes and gene products from the following aspects: biological process (BP), molecular function (MF), and cellular component (CC) [17]. Using the clusterProfiler package (http://bioconductor.org/packages/release/bioc/html/clusterProfiler.html) [18] in R, the key prognostic genes were performed GO functional enrichment analysis. The terms with q-value < 0.05 were selected as the significant functions.

### Multivariate survival analysis

To examine the overall influence of the key prognostic genes on prognosis, multivariate survival analysis was conducted for the key prognostic genes. Besides, Receiver Operating Characteristic (ROC) curve was drawn using the survivalROC package [19] in R.

### Validation of the key prognostic genes using an independent microarray data

To confirm that the repeatability and portability of key prognostic genes, microarray data of GSE17260 were downloaded from Gene Expression Omnibus (GEO, https://www.ncbi.nlm.nih.gov/geo/) and used for survival analysis. GSE17260, which was sequenced on the platform of Agilent-014850 Whole Human Genome Microarray 4x44K G4112F, included a total of 110 serous OC samples. Using the survival package [20] in R, cox regression analysis was conducted for the microarray data.

## Results

### Data preprocessing, cluster analysis and differential analysis

**Fig. 1:**
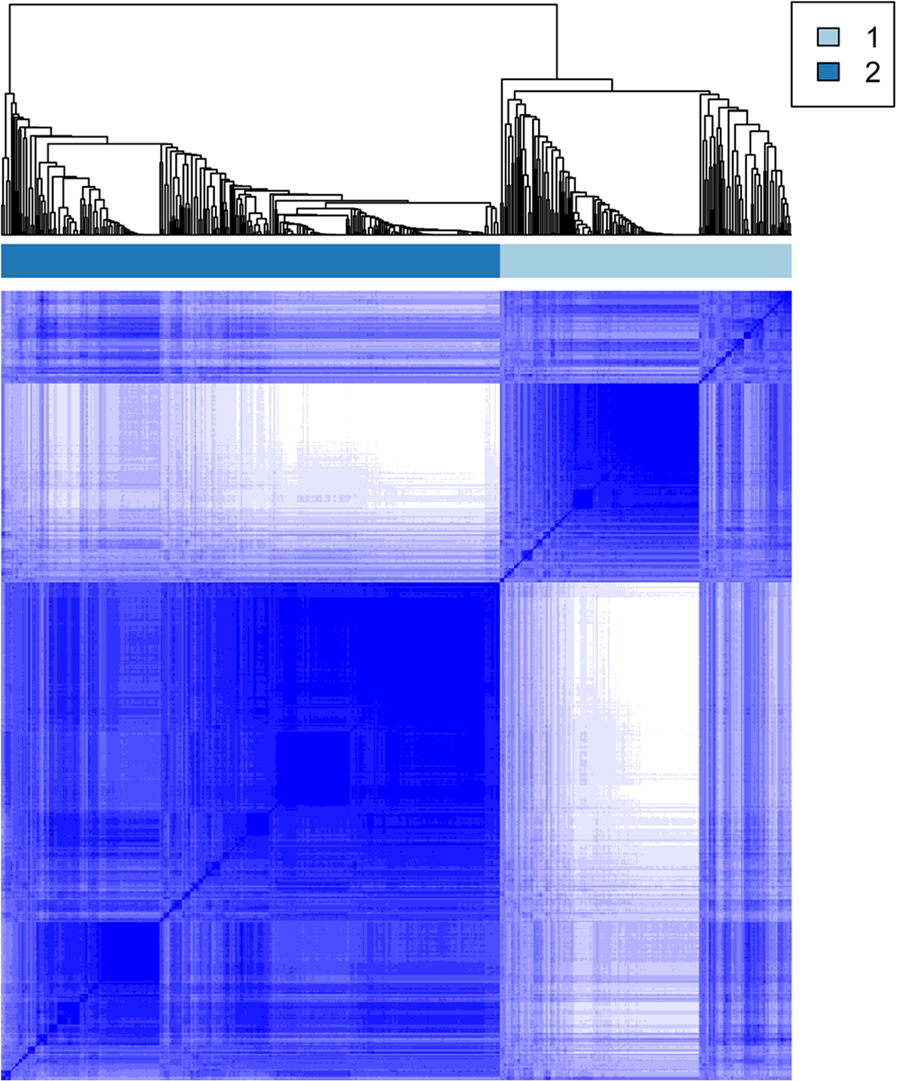
The cluster heatmap for the 8873 genes

Through data preprocessing, the expression values of a total of 12042 genes were acquired. After screening the primary OC tissues with clinical information, a total of 564 samples were obtained. The variance and median of gene expression levels were calculated, and then the genes with little expression changes among the samples were removed. After that, a total of 8873 genes were screened. The expression levels of the 8873 genes in the 564 samples were performed centralization. Then, the 564 samples were pre-classified into two groups (one group had 195 samples and the other group had 369 samples) through cluster analysis to identify the prognosis difference among all samples. The heatmap of cluster analysis showed that the 8873 genes could distinguish different samples (Fig. 1). Subsequently, a total of 3668 genes were identified as feature genes using differential analysis.

### Screening of stable feature genes and key prognostic genes

**Fig. 2:**
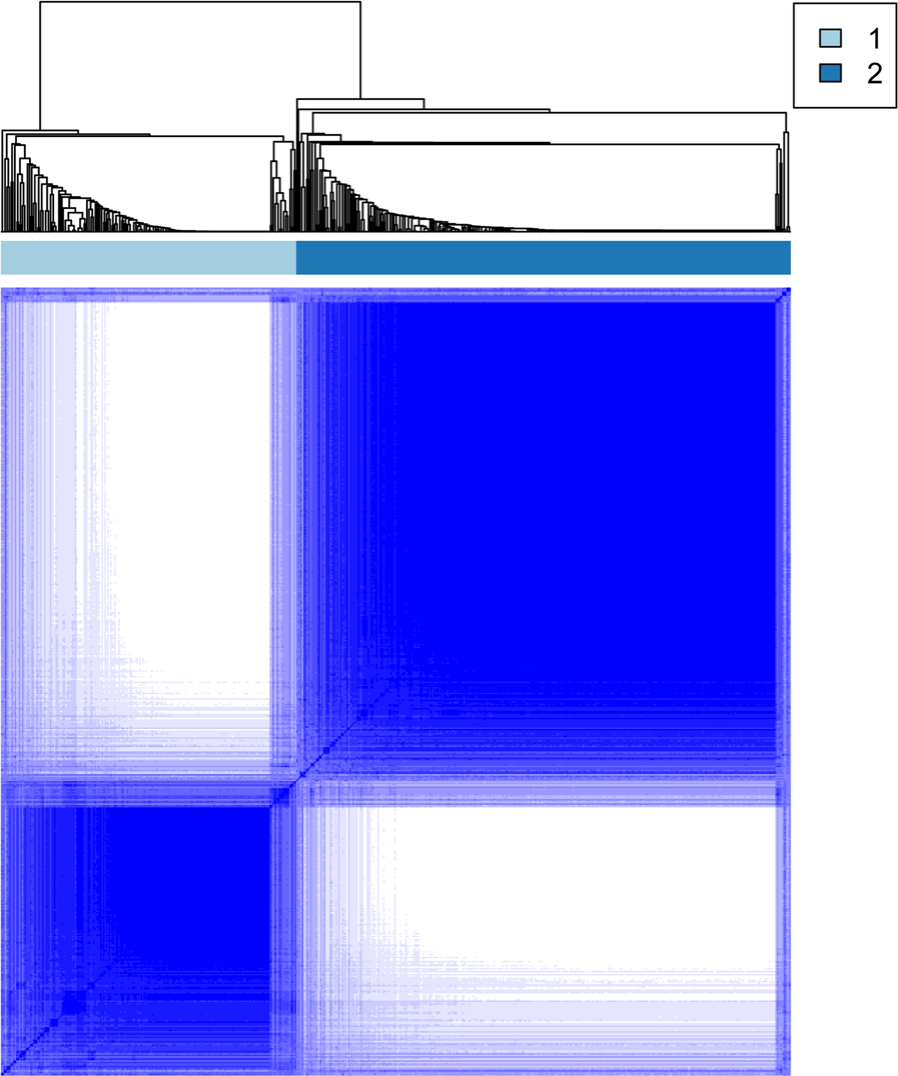
The cluster heatmap for the 3393 stable feature genes

Through the loop iteration of the expression levels of feature genes, a total of 3393 stable feature genes were obtained. The two clusters with different prognosis status of the stable feature genes included 211 samples and 353 samples, respectively. The samples under the same cluster exhibited high correlation (Fig. 2). The clinical features of the two sample groups were further observed, and the result showed that the two group (cluster 1 and cluster 2) samples had significant differences in both the stage (Table 1A) and the grade (Table 1B).

**Table 1:** The stage and grade distribution of the two groups of samples divided by the 3393 stable feature genes

A Cluster	StageIII	StageIV	Total number of samples	Fisher’s exact *p*-value
Cluster1	144	53	197	4.699e-06
Cluster2	260	31	291	
B Cluster	G2	G3	Total number of samples	Fisher’s exact *p*-value
Cluster1	10	196	206	8.491e-06
Cluster2	59	279	338	

**Fig. 3:**
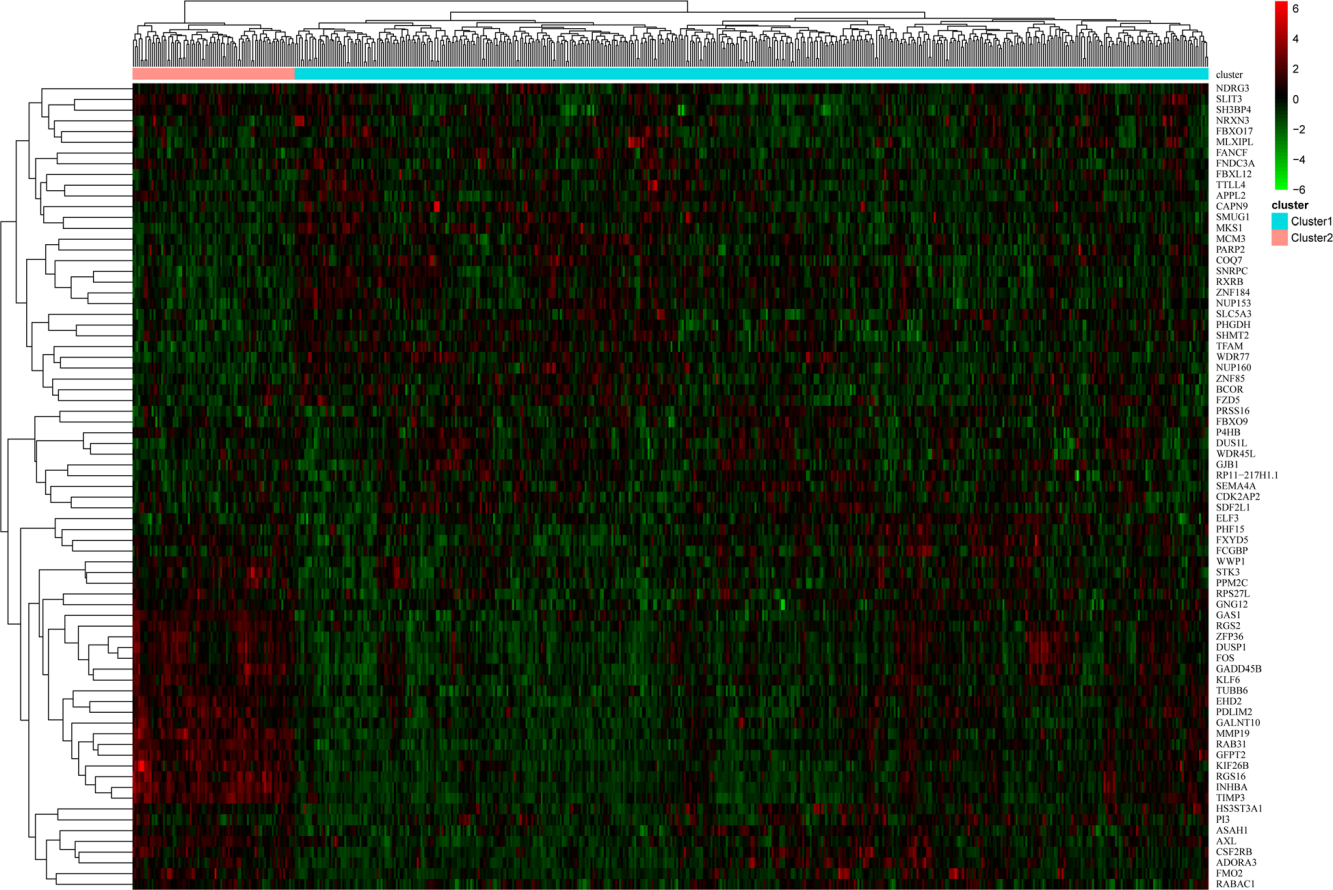
The cluster heatmap for the 75 prognostic seed genes

**Fig. 4:**
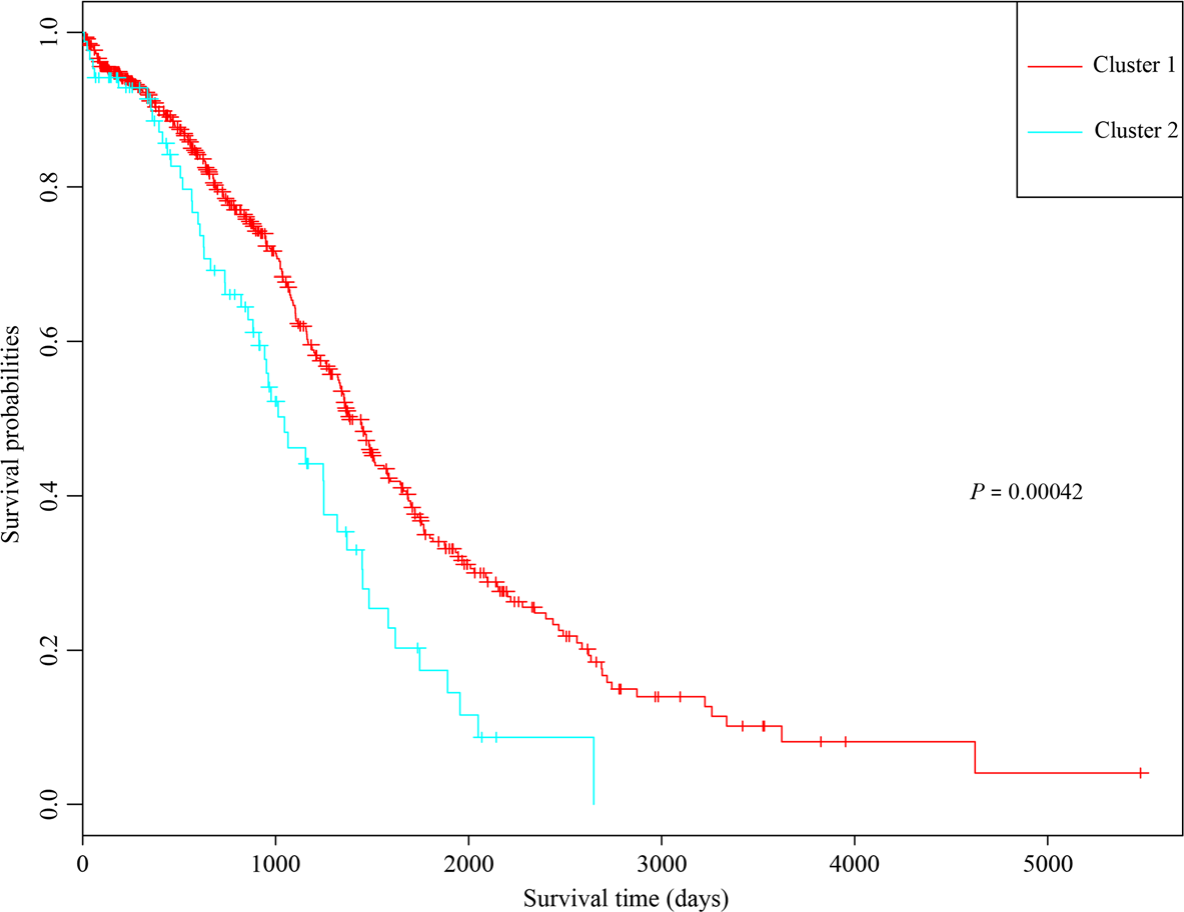
The result of survival analysis for the two groups of samples divided by the 75 prognostic seed genes

Using KM survival analysis, a total of 75 prognostic seed genes were identified. Afterwards, cluster analysis was conducted for the prognostic seed genes, and the cluster heatmap suggested that the samples could be obviously divided into two groups (one group had 479 samples and the other group had 85 samples) (Fig. 3). Using log-rank test, the survival analysis for the two group samples showed that they had significant differences in prognosis (Fig. 4), indicating that the prognostic seed genes could classify the samples in prognostic level.

The cluster heatmap showed that the expression levels of some genes among the 75 prognostic seed genes were not very obvious, thus the key prognostic genes were further extracted. After trying different log_2_ FC value for screening gene set, we found that |log_2_FC| > 0.7 was the most optimal threshold. Under |log_2_FC| > 0.7, the gene set containing 25 genes had the most significant influence on prognosis, as they can distinguish patients with different survival status. Thus, the 25 genes were selected as the key prognostic genes, including AXL receptor tyrosine kinase (*AXL*), FBJ murine osteosarcoma viral oncogene homolog (*FOS*), Kruppel-like factor 6 (*KLF6*), WD repeat domain 77 (*WDR77*), dual specificity phosphatase 1 (*DUSP1*), growth arrest and DNA damage inducible beta (*GADD45B*), and slit guidance ligand 3 (*SLIT3*) (Table 2).

**Table 2:** The information of the 25 key prognostic genes

Gene symbol	Gene Name
*AXL*	AXL receptor tyrosine kinase
*EHD2*	EH domain containing 2
*FOS*	FBJ murine osteosarcoma viral oncogene homolog
*KLF6*	Kruppel-like factor 6
*MKS1*	Meckel syndrome, type 1
*PDLIM2*	PDZ and LIM domain 2
*RAB31*	RAB31, member RAS oncogene family
*TIMP3*	TIMP metallopeptidase inhibitor 3
*WDR77*	WD repeat domain 77
*ZFP36*	ZFP36 ring finger protein
*CSF2RB*	colony stimulating factor 2 receptor beta common subunit
*DUSP1*	dual specificity phosphatase 1
*FMO2*	flavin containing monooxygenase 2
*GFPT2*	glutamine-fructose-6-phosphate transaminase 2
*GADD45B*	growth arrest and DNA damage inducible beta
*GAS1*	growth arrest specific 1
*INHBA*	inhibin beta A
*KIF26B*	kinesin family member 26B
*MMP19*	matrix metallopeptidase 19
*GALNT10*	polypeptide N-acetylgalactosaminyltransferase 10
*RGS16*	regulator of G-protein signaling 16
*RGS2*	regulator of G-protein signaling 2
*STK3*	serine/threonine kinase 3
*SLIT3*	slit guidance ligand 3
*TUBB6*	tubulin beta 6 class V

### Functional enrichment analysis

With q-value < 0.05 as the threshold, the 25 key prognostic genes were significantly enriched in 14 terms. The enriched functions mainly included ovulation cycle (q-value = 0.004227, which involved *AXL* and *SLIT3*), reproductive structure development (q-value = 0.004227), and regulation of reproductive process (q-value = 0.004227) (Table 3).

**Table 3:** The GO (Gene Ontology) functions enriched for the 25 key prognostic genes

Term	Description	Gene symbol	Q-value
GO:0042698	ovulation cycle	*INHBA*, *MMP19*, *SLIT3*, *AXL*	0.004227
GO:0048608	reproductive structure development	*INHBA*, *MMP19*, *WDR77*, *STK3*, *SLIT3*, *AXL*	0.004227
GO:2000241	regulation of reproductive process	*INHBA*, *WDR77*, *STK3*, *DUSP1*	0.004227
GO:0061458	reproductive system development	*INHBA*, *MMP19*, *WDR77*, *STK3*, *SLIT3*, *AXL*	0.004227
GO:0046660	female sex differentiation	*INHBA*, *MMP19*, *SLIT3*, *AXL*	0.004227
GO:0001554	luteolysis	*MMP19*, *SLIT3*	0.004227
GO:1901654	response to ketone	*KLF6*, *SLIT3*, *DUSP1*, *FOS*	0.009043
GO:0097305	response to alcohol	*INHBA*, *KLF6*, *SLIT3*, *DUSP1*, *FOS*	0.012039
GO:0022602	ovulation cycle process	*INHBA*, *MMP19*, *SLIT3*	0.024753
GO:0051591	response to cAMP	*MMP19*, *DUSP1*, *FOS*	0.025054
GO:0030728	ovulation	*INHBA*, *MMP19*	0.027454
GO:0008585	female gonad development	*INHBA*, *MMP19*, *SLIT3*	0.027454
GO:0046545	development of primary female sexual characteristics	*INHBA*, *MMP19*, *SLIT3*	0.028787
GO:0007548	sex differentiation	*INHBA*, *MMP19*, *SLIT3*, *AXL*	0.030429

### Multivariate survival analysis

**Fig. 5:**
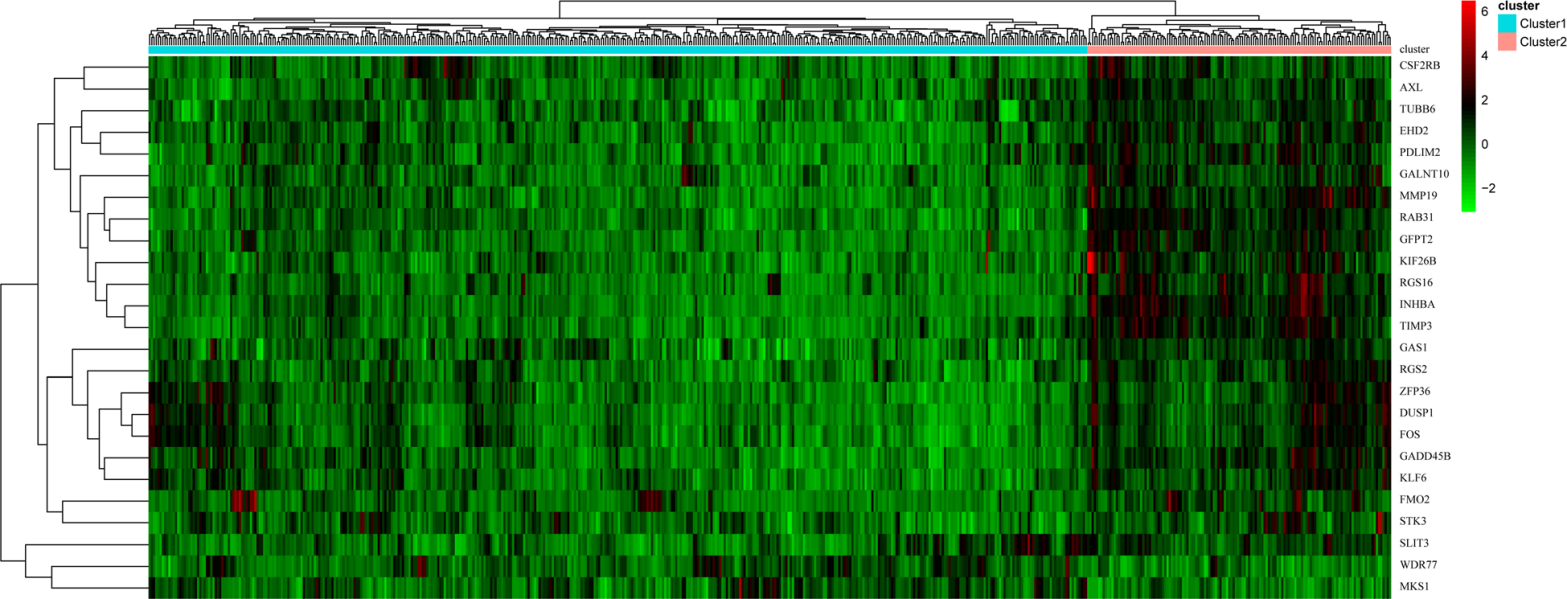
The cluster heatmap for the 25 key prognostic genes

**Fig. 6:**
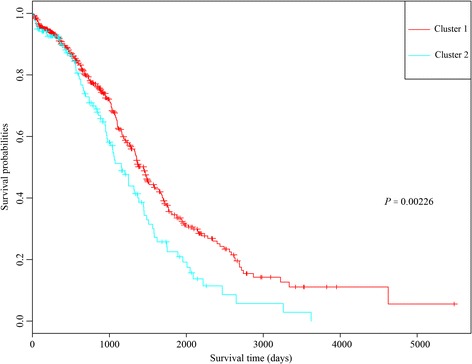
The Kaplan-Meier (KM) survival curve for the two groups of samples divided by the 25 key prognostic genes

The cluster heatmap of the 25 key prognostic genes suggested that these genes could divide the samples into two groups (Fig. 5). The multivariate survival analysis for the key prognostic genes showed that the overall survival time of the patients in the two groups had significant difference (*p-*value = 0.00226) (Fig. 6). Therefore, the 25 key prognostic genes could effectively differentiate the samples and were significantly associated with prognosis.

### Validation of the key prognostic genes using an independent microarray data

**Fig. 7:**
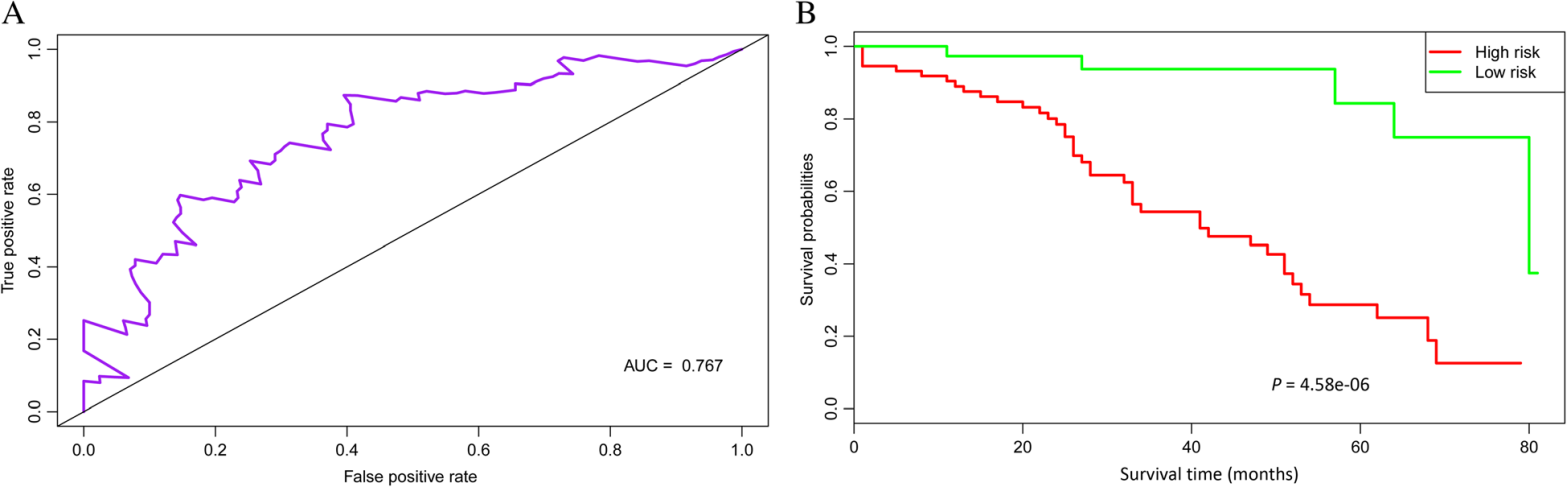
The Receiver Operating Characteristic (ROC) curve (**a**) and Kaplan-Meier (KM) survival curve (**b**) of multivariate survival analysis for the validation dataset. The results showed that the 25 key prognostic genes also had good classification effects for the validation dataset

The microarray data of GSE17260 were taken as validation dataset to confirm that the repeatability and portability of the key prognostic genes. Multivariate survival analysis showed that the 25 key prognostic genes also had good classification effects for the validation dataset (Fig. 7). This suggested that the 25 key prognostic genes were key genes affecting the prognosis of OC.

## Discussion

In this study, a total of 564 samples were obtained from data preprocessing, which were pre-classified into two groups. Afterwards, differential analysis identified 3668 feature genes. Besides, 3393 stable feature genes were obtained through loop iteration, and 75 genes among them were identified as prognostic seed genes. Moreover, 25 prognostic seed genes were selected as the key prognostic genes (including *AXL*, *FOS*, *KLF6*, *WDR77*, *DUSP1*, *GADD45B*, and *SLIT3*). Multivariate survival analysis indicated that the 25 key prognostic genes could effectively differentiate the samples and were significantly associated with prognosis. In addition, the microarray data of GSE17260 further confirmed that the key prognostic genes were key genes affecting the prognosis of OC.

GADD45 proteins mediate many cellular functions such as cell cycle control, genotoxic stress, DNA repair, and senescence, additionally, GADD45 proteins functions as tumor suppressors through their pro-apoptotic activities [21, 22]. Overexpression of *GADD45A* may be implicated in the pro-apoptosis effect of the synthetic retinoid CD437 on ovarian cancer cells [23]. Oliveira-Ferrer et al. deem that *c-FOS* may affect OC progression through exerting pro-apoptotic effect and altering peritoneal adhesion of OC cells [24]. Mahner et al. find that down-regulated *c-Fos* plays a role in tumor progression in OC and *c-Fos* may be used as prognostic factor for the disease [25]. Furthermore, *KLF6* and its alternative splicing isoform *KLF6-SV1* are related to the main clinical characteristics of EOC, thus they may serve as therapeutic targets for changing the development and dissemination of OC [26]. Above evidence declared that *GADD45B*, *FOS*, and *KLF6* might be correlated with the prognosis of OC.

Ligr et al. find that *p44*/*Mep50*/*WDR77* is associated with hormone effects during ovarian tumorigenesis [27]. Via regulating the p38 MAPK-mediated p-glycoprotein overexpression, *DUSP1* may cause the resistance of human OC cells to paclitaxel [28, 29]. The glucocorticoid administration to OC patients is correlated with increased expression of map kinase phosphatase 1 (*MKP1*, also known as *DUSP1*) and serum and glucocorticoid-regulated kinase 1 (*SGK1*), indicating that glucocorticoids may weaken chemotherapy effect in OC patients by promoting the expression of anti-apoptotic genes [30]. Additionally, *MKP1* can be induced by cisplatin via ERK signaling-associated phosphorylation, and the ERK-MKP1 signaling functions in overcoming cisplatin resistance in OC patients [31]. Thus, *WDR77* and *DUSP1* might play roles in the development of OC.

Previous study find that Growth arrest-specific gene 6 (GAS6)/AXL pathway has an influence on the complex events occurring during the early stage of OC [32]. GAS6/AXL targeting can be an effective mean for inhibiting the progression of metastatic OC, and the soluble AXL receptor is a promising agent for treating the disease [33]. Since cortisol suppressing SLIT/Roundabout (ROBO) pathway contributes to the regeneration of ovarian surface epithelium, the pathway may be a target for controlling the SLIT/ROBO system in OC [34, 35]. Qiu et al. demonstrate that *SLIT2* can serve as tumor suppressor in OC, thus it may be used as a promising therapeutic target for the disease [36]. Functional enrichment analysis showed that *AXL* and *SLIT3* were enriched in ovulation cycle, suggesting that *AXL* and *SLIT3* might also be involved in OC through affecting ovulation cycle.

## Conclusions

In conclusion, a total of 3668 feature genes and 25 key prognostic genes were screened by bioinformatics analysis. Besides, several key genes (*AXL*, *FOS*, *KLF6*, *WDR77*, *DUSP1*, *GADD45B*, and *SLIT3*) might be associated with the prognosis of OC. However, the functions of these key genes need to be confirmed by experimental researches in future.

## Abbreviations

AXL: AXL receptor tyrosine kinase
BP: Biological process
CC: Cellular component
CXCR4: Chemokine receptor 4
DUSP1: Dual specificity phosphatase 1
EOC: Epithelial ovarian cancer
FC: Fold change
FOS: FBJ murine osteosarcoma viral oncogene homolog
FOXM1: Forkhead box M1
GADD45B: Growth arrest and DNA damage inducible beta
GAS6: Growth arrest-specific gene 6
GEO: Gene Expression Omnibus
GO: Gene Ontology
HMGA2: High mobility group A2
KLF6: Kruppel-like factor 6
KM: Kaplan-Meier
MF: Molecular function
MKP1: Map kinase phosphatase 1
OC: Ovarian cancer
ROC: Receiver Operating Characteristic
SGK1: Glucocorticoid-regulated kinase 1
SLIT3: Slit guidance ligand 3
TCGA: The Cancer Genome Atlas
TEAD4: TEA domain family member 4
WDR77: WD repeat domain 77
YAP: Yes-associated protein 1
